# Information status relates to production, distribution, and comprehension

**DOI:** 10.3389/fpsyg.2013.00235

**Published:** 2013-05-10

**Authors:** Jennifer E. Arnold

**Affiliations:** Department of Psychology, University of North Carolina at Chapel HillChapel Hill, NC, USA

MacDonald's (2013) paper makes two strong contributions to the psycholinguistics literature, in my opinion. First, it calls for a serious consideration of how cognitive pressures affect production processing, which is a necessary step for the development of mechanistic theories of language use. A second major contribution of the PDC is that it provides a theoretical explanation for the body of evidence showing that language comprehension is affected by the user's experience, for example where frequent structures and words are processed more easily than less frequent structures and words. The boldest portion of this proposal is that processing constraints on production are the primary source of typological distributions, which in turn affect comprehension biases. This is exactly the kind of theoretical claim that helps the field move forward, by being clear, simple, and far-reaching.

At the same time, I argue that the PDC would benefit from greater consideration of information status. Language production is not primarily the retrieval of words and structures. Instead, these activities serve the speaker's goal, which is to communicate a meaning to the addressee. Likewise, linguistic meanings consist of more than identifying who did what. An important part of communication involves linking the utterance to information in the world and the linguistic context. Information varies across dimensions such as given vs. new, and topic vs. focus (for a review see Arnold et al., 2013).

Information status is critical to understanding the PDC's proposed mechanisms, for two reasons. First, it is intimately related to the memory/attention cognitive mechanisms that are central to the reasoning behind the PDC. Second, these correlations highlight an important challenge for the PDC, which is to specify the distributional patterns that comprehenders use. I will illustrate these points with evidence of how information status is related to both (1) acoustic prominence and disfluency, and (2) syntactic structure and animacy.

## Information status, production difficulty, and acoustic reduction

Information status is important for the PDC, because it is intimately related to both linguistic form and production difficulty. Much of my recent work has focused on this question with respect to acoustic prominence and disfluency. A well-established pattern is for speakers to use acoustically reduced (e.g., unaccented and/or shorter pronunciations) words for information that is given or in focus, while longer, prominent pronunciations are used for new or unpredictable information (Halliday, 1967; Fowler and Housum, 1987). Like word order, acoustic variation also varies as a function of production difficulty. For example, when speakers produce disfluencies like “uh,” “um,” or repeated words, other words in the vicinity are lengthened (Bell et al., 2003). Likewise, when planning is facilitated or occurs earlier, word duration is shorter (Christodoulou, 2012; Gillespie, 2011), and planning difficulty can be associated with higher pitch and longer duration (Christodoulou, 2009).

Of particular importance to the PDC, information status itself is correlated with planning difficulty, as evidenced by its relation to disfluency (Arnold and Tanenhaus, 2011), and utterance initiation time (Kahn and Arnold, 2012). Given information is simply easier to talk about than new information. This has led to the proposal that planning and production constraints themselves contribute to the acoustic patterns associated with information status (Kahn and Arnold, 2012; Arnold and Watson, under review), and reference production more generally (Arnold, 2010). This claim is supported by evidence that reduction is greater when a word is facilitated both lexically and conceptually, as opposed to just conceptually (Kahn and Arnold, 2012). Given discourse status is also associated with predictability, which affects acoustic reduction in and of itself (e.g., Lam and Watson, 2010).

### Acoustic variation: implications for comprehension

On one hand, this work suggests that the PDC is on the right track, and could even be expanded to include non-syntactic linguistic form. On the other hand, it raises questions about what is being learned. If the PDC is right—and if it extends to things like acoustic variation—the distribution of acoustically prominent forms should translate into comprehension facilitation in the contexts where particular forms are expected. Yet disfluency and acoustic prominence are correlated with multiple informational properties, and listeners appear to use fine-grained calculations of distributions in only some cases.

For example, consider the effects that disfluency has on the comprehension of definite noun phrases. When a listener hears *Click on theee, uh…*, comprehension is facilitated if the object mentioned is either new to the discourse (Arnold et al., 2004), or an unusual, unfamiliar object (Arnold et al., 2007). Both of these patterns reflect production biases, in that speakers are more likely to be disfluent when mentioning new or unfamiliar things—both of which are likely to cause production difficulty (Arnold and Tanenhaus, 2011). What, then, do language users learn? Do they link disfluency with newness, unfamiliarity, or a more general category of “things that are difficult to refer to”? Even though “reference difficulty” does not seem to be a ready-made category, Arnold et al. (2007) present evidence that the unfamiliar bias can be disrupted when the speaker is expected to have difficulty recognizing objects. This suggests that comprehenders can keep track of production difficulty, and moreover that they can do so contingent on a particular speaker or situation.

A similar question applies to the comprehension of acoustic prominence. Eyetracking studies have shown that listeners use acoustic prominence extremely rapidly to form biases about the word's referent (Dahan et al., 2002; Arnold, 2008). That is, an acoustically prominent word (e.g., *BACON*) is initially assumed to have a previously unmentioned (discourse-new) referent. Yet even though information status correlates with difficulty, difficulty can also stem from other sources, like distraction. That is, sometimes a prominent *BACON* reflects discourse-newness, and sometimes a prominent *BAYY-CON* might indicate that the speaker is distracted, regardless of discourse status. Thus, different kinds of prominence are likely correlated with different situations. If listeners can remember fine-grained contingent distinctions, they should not assign a discourse-new interpretation to a word that is long in a distracted context. However, in a recent study we found that hearing distracted speech led to an increase in the discourse-new bias overall, even for relatively reduced words (Arnold et al., 2012). This experiment has implications for the PDC, as it suggests that listeners categorize input stimuli in ways that do not preserve all the contingent properties of that input.

## Information status, animacy, and syntactic structure

The PDC acknowledges some effects of information/discourse status in its description of the production and planning constraints that affect language form. In MacDonald, section 3.1 points out that production facilitation is related to given or salient discourse status and conceptual familiarity. Thus, the facilitation related to given discourse status is one of the components of MacDonald's argument that Easy First guides production.

My argument here is that information status deserves greater consideration within the PDC, because it is related not only to Easy First, but also to virtually every other component of the PDC. Information status is not just “one more constraint” on production, but is arguably one of the earliest constraints affecting sentence production. Sentences are typically produced in the context of discourses, which connect entities and events through anaphoric references. Information status may also encourage Plan Reuse, given the tendency for speakers to produce parallel structures in which referents fill the same semantic and information-structure roles (Arnold, 1998).

Critically, information status is also likely to be correlated with properties like animacy, which plays a central role in the PDC. People tend to be interested in the actions of animate beings, and discourses are naturally organized around the goals and interests of the interlocutors. Thus, it stands to reason that discourse topics are animate more often than not.

On one hand, the correlation between animacy and information status shores up one of the main tenets of the PDC, which is that linguistic form and meaning are correlated with production difficulty. On the other hand, this leads to a critical challenge for the PDC, which is to define the ways in which these distributions are acquired and used in comprehension.

### Information status in the PDC: distribution and comprehension

The questions that arise for disfluency and acoustic prominence have parallels in the syntactic comprehension findings that are at the center of the PDC. The PDC provides a compelling framework for linking production and comprehension processing. However, as it stands, the details about what exactly is learned need to be fleshed out. This problem stems from one of the foundational observations of the PDC, which is that language form is massively correlated with conceptual, informational, and psychological properties.

MacDonald and colleagues have addressed this issue as the “grain” problem, and have suggested that comprehenders may learn correlations at many different grains (e.g., Wells et al., 2009). Indeed, evidence suggests that language users can represent fine-grained contingent patterns of distributional information. For example, MacDonald and Thornton (2009) found that local modification is only preferred in cases where the intervening material is long, as in *Mary likes it when the dolphins at Sea World are swimming {very slowly/ very much*.} This is connected to the tendency for speakers not to produce long-before-short orders. Likewise, in example 2, MacDonald points out that RCs headed by animates are not expected to be object RCs, because that pattern is rare.

At the same time, some contingencies in the input are not preserved. Learning any distributional pattern requires language users to abstract away from individual tokens in some meaningful way, generalizing over the input. In production, too, MacDonald predicts that one structure can inherit a bias from other structures, for example in that the rate of passive relatives in a language varies with respect to the rate of passives in other structures.

Thus, a challenge for the PDC is to identify specific ways in which people retain sensitivity to contingencies in the input—as well as ways in which they do not. This problem is complicated by the systematic correlation between information status and every level of the PDC: production difficulty, linguistic form, and conceptual categories like animacy. Consider MacDonald's example 5a, *The boy/toy that the girl splashed….* Animate nouns tend not to occur as the head of object relative clauses, while inanimate nouns do. In keeping with this distributional pattern, comprehension of object relatives is easier when the head noun is inanimate. These findings suggest that listeners have learned the correlation between animacy and syntactic structure. Yet animates also tend to be “conceptually and topically salient” (Gennari et al., 2012, p.152). Instead of learning that object relatives pattern with inanimates, comprehenders may instead learn that they pattern with non-topical things, or with psychological properties associated with topicality, like attention. Given the importance of production difficulty to the PDC, it is even possible that users represent information in terms of “ease of production,” and its relationship to syntactic structure.

Why does it matter whether listeners are learning that relative clauses pattern with animacy, or something else that is closely related? Most fundamentally, this question addresses the PDC's stated goal of developing mechanistic accounts of language processing. The PDC hinges on the idea that existing distributional patterns are learned and used for comprehension. The details about what is learned are also critical for assessing the claim that production processes constrain the learning process more than comprehension processes (MacDonald, section 3.4). It is quite possible that some patterns are more salient, or easier to learn (Amato and MacDonald, 2010). Thus, it is necessary to assess the degree to which comprehension biases guide the acquisition of some grains, but not others.

## Conclusions

MacDonald's Production-Distribution-Comprehension theory provides a far-reaching and provocative hypothesis. I have proposed that the theory would be even stronger with a detailed consideration of information status. Work from my lab and others has shown how production difficulty is associated with both information status and linguistic form, which supports MacDonald's claim that production processing guides language form. Identifying how language users represent and use these patterns is critical for assessing the claims of the PDC.
